# Eukaryotic Parasites Are Integral to a Productive Microbial Food Web in Oxygen-Depleted Waters

**DOI:** 10.3389/fmicb.2021.764605

**Published:** 2022-01-06

**Authors:** Elizabeth A. Suter, Maria Pachiadaki, Gordon T. Taylor, Virginia P. Edgcomb

**Affiliations:** ^1^Biology, Chemistry & Environmental Studies Department, Center for Environmental Research and Coastal Oceans Monitoring, Molloy College, Rockville Centre, NY, United States; ^2^School of Marine and Atmospheric Sciences, Stony Brook University, Stony Brook, NY, United States; ^3^Department of Biology, Woods Hole Oceanographic Institution, Woods Hole, MA, United States; ^4^Department of Geology & Geophysics, Woods Hole Oceanographic Institution, Woods Hole, MA, United States

**Keywords:** 18S (SSU) rRNA gene, oxygen-depleted environment, oxygen minimum zone (OMZ), protist, Syndiniales, parasite, eukaryotes, network analysis

## Abstract

Oxygen-depleted water columns (ODWCs) host a diverse community of eukaryotic protists that change dramatically in composition over the oxic-anoxic gradient. In the permanently anoxic Cariaco Basin, peaks in eukaryotic diversity occurred in layers where dark microbial activity (chemoautotrophy and heterotrophy) were highest, suggesting a link between prokaryotic activity and trophic associations with protists. Using 18S rRNA gene sequencing, parasites and especially the obligate parasitic clade, Syndiniales, appear to be particularly abundant, suggesting parasitism is an important, but overlooked interaction in ODWC food webs. Syndiniales were also associated with certain prokaryotic groups that are often found in ODWCs, including Marinimicrobia and Marine Group II archaea, evocative of feedbacks between parasitic infection events, release of organic matter, and prokaryotic assimilative activity. In a network analysis that included all three domains of life, bacterial and archaeal taxa were putative bottleneck and hub species, while a large proportion of edges were connected to eukaryotic nodes. Inclusion of parasites resulted in a more complex network with longer path lengths between members. Together, these results suggest that protists, and especially protistan parasites, play an important role in maintaining microbial food web complexity, particularly in ODWCs, where protist diversity and microbial productivity are high, but energy resources are limited relative to euphotic waters.

## Introduction

Oxygen-depleted water columns (ODWCs) are geographically limited regions of the global ocean where disproportionately large biogeochemical turnover of essential elements, like nitrogen, occurs (Codispoti et al., 2001; Arrigo, 2005). Much of this turnover is mediated by unique prokaryotic microbial communities (Wright et al., 2012). The permanently anoxic Cariaco Basin on Venezuela’s northern coast represents an endmember among ODWCs; a shallow sill prevents exchange with oxygenated open ocean Caribbean Sea water, and oxygen begins to decline at about 100–200 m below the sea surface (Richards, 1975). From about 350 m to the bottom (1380 m), conditions are permanently anoxic and reduced compounds such as sulfide, ammonium, and methane accumulate toward the seabed (Scranton, 1988; Scranton et al., 2014). Established gradients are similar to those observed in open ocean oxycline and oxygen minimum zone ecosystems, as well as euxinic ecosystems, such as the Black Sea, fjords, meromictic lakes, and anoxic sediments.

Microbial communities occupying the Cariaco Basin’s oxycline and anoxic zone are similar to those of other ODWCs. Prokaryotes involved in carbon, nitrogen, and sulfur transformations are enriched at midwater depths (Madrid et al., 2001; Lin et al., 2006; Rodriguez-Mora et al., 2013; Suter et al., 2018). Activities, such as chemoautotrophy (dark carbon fixation), heterotrophy, ammonia oxidation, and denitrification are all enhanced at these midwater depths where both oxygen and sulfide concentrations are low (Taylor et al., 2001; Cernadas-Martín et al., 2017; Suter et al., 2020). In the underlying euxinic layer, unique prokaryotic communities resemble those of anoxic sediments and have been linked to processes such as fermentation, methanogenesis, and sulfate reduction (Madrid et al., 2001; Suter et al., 2018).

Previous work on Cariaco Basin protist communities reveal similar enrichments in cell abundances at midwater depths, and a transition in community composition along this redox gradient. Flagellated protists consistently exhibit localized abundance peaks between 250 and 450 m (Taylor et al., 2001, 2006). The first application of 18S rRNA sequencing to an anoxic marine community was in the Cariaco Basin, and revealed, at the time, novel eukaryotic lineages unique to this site (Stoeck et al., 2003), which have since been identified in permanently stratified fjords with redoxclines (Edgcomb and Pachiadaki, 2014) and methane seep sediments (Pasulka et al., 2016). Later, clone libraries and 454 pyrosequencing revealed a high diversity of protist communities that changed dramatically in composition over the redox gradient and exhibited a degree of vertical endemism, likely controlled by stratified geochemical conditions (Edgcomb et al., 2011a; Orsi et al., 2011). Furthermore, these studies revealed that the Cariaco Basin’s euxinic waters were occupied by a previously undiscovered class of ciliates (Cariacotrichea) that has subsequently been reported for other ODWCs (Orsi et al., 2012b).

Previous protist community studies from ODWCs have primarily focused on the roles of biotic interactions such as predation and symbioses. Predation by protists has been shown to be a major control on prokaryotic community composition. For example, bacterial richness decreased in predator-exclusion experiments conducted with samples from Cariaco’s midwater depths (Lin et al., 2007). Symbioses under anoxic conditions have also been well documented, particularly between ciliates and prokaryotes (Edgcomb et al., 2011b; Edgcomb and Pachiadaki, 2014). Parasitism is more poorly documented in anaerobic marine protist assemblages. However, there is a growing appreciation for a ubiquitous marine parasite taxon, Syndiniales (phylum Dinoflagellata), which includes diverse sub-groups (Guillou et al., 2008), and is widely distributed throughout the ocean (de Vargas et al., 2015; Anderson and Harvey, 2020). For example, the Tara Oceans project, which surveyed the tropical epipelagic and mesopelagic ocean, found that parasitic protist groups accounted for up to 59% of SSU rRNA gene richness and approximately 53% of abundance of pico-nanoplankton (0.8–5.0 μm). Eighty-nine percent of the parasitic sequences accounting for this pico-nanoplankton diversity and abundance were affiliated with Groups I and II Syndiniales (de Vargas et al., 2015). Exploring the bathypelagic global ocean, the Malaspina-2010 Expedition found that Group II Syndiniales were one of the most highly represented protist groups in the majority of samples (Pernice et al., 2016). Signatures of Syndiniales have also been detected in several ODWCs (Parris et al., 2014; Duret et al., 2015; Torres-Beltrán et al., 2018; De la Iglesia et al., 2020).

Historically, microbial interactions such as parasitism were studied using direct observational methods like microscopy (e.g., Chambouvet et al., 2008, 2011). While such methods are important for confirming precise host–parasite relationships, they are impractical for determining the potentially vast array of biotic interactions that occur among the vast number of individual members within planktonic communities. For example, estimates of only picoplankton in the ocean range from 10^5^ to 10^7^ cells per liter (Massana, 2011). With increasing use of next generation sequencing (NGS) data, ecologists have new tools to discover potential biotic interactions from complex communities. For example, Käse et al. (2021) were able to recognize host-parasite relationships previously described by microscopy using an 18S rRNA gene dataset from a long-term ecological research site in the North Sea. NGS data also enable investigation of relationships using approaches such as network analysis, which portray positive and negative associations between amplicon sequence variants (ASVs) or operational taxonomic units (OTUs) across broad temporal and spatial domains. Network analyses are increasingly applied to 16S or 18S rRNA datasets to determine potential interactions, such as cross-feeding, competition, cooperation, grazing, parasitism, or simply to identify taxa with similar nutritional needs or specialized niches (Steele et al., 2011; Banerjee et al., 2018). Targeted cultivation experiments have recently confirmed several relationships that were first inferred from a human microbiome network analysis (Tipton et al., 2018), validating network analysis as a tool for deciphering potential biotic relationships. In this study, we apply network analysis among other statistical techniques to describe potential interactions within Cariaco Basin microbiomes, with special focus on parasites. This analysis is one of the few from ODWCs to examine biotic relationships among all domains (Bacteria, Archaea, Eukarya) and highlights inter-trophic level interactions in this complex and productive anaerobic ocean food web.

## Materials and Methods

### Sample Collection

All samples were collected aboard the R/V *Hermano Ginés* in the coastal waters of Venezuela at the CARIACO Ocean Time-Series station (10.51°N, 64.67°W^1^). Samples for microbial community analysis were obtained during two oceanographic cruises, May 7–9, 2014 and November 5–7, 2014, corresponding to 2 legs of the CARIACO Ocean Time Series project: CAR212 and CAR216, respectively. Our group previously published on prokaryotic community ecology (Suter et al., 2018) and microbial nitrogen cycling (Suter et al., 2020) using contemporaneous samples from these expeditions, and sample collection and processing are explained in detail in those studies. Briefly, seawater samples for DNA metabarcoding were collected from 6 depths spanning the redox gradient during both cruise legs. Seawater was collected by Niskin bottles mounted on a rosette, equipped with a SeaBird SBE 25 CTD (conductivity-temperature-depth) sensor package, a SeaBird SBE 43 dissolved oxygen sensor, and a Sea Tec c-beam transmissometer (660 nm) to measure beam attenuation. Beam attenuation was previously shown to be an indicator for the microbial abundance peak consistently observed near the oxic-anoxic boundary (Taylor et al., 2001). The sampling scheme during each cruise targeted this layer, as well as features above and below it. Additional environmental variables including sulfide, ammonium, nitrate, nitrite, phosphate, microbial abundance, flagellate abundance, particulate sulfur, total zero-valent sulfur, and rates of chemoautotrophic and heterotrophic activity were collected during separate legs of each cruise on May 12, 2014 and November 10, 2014, respectively. The results from these biogeochemical measurements are described thoroughly in Suter et al. (2018) and analytical methods were those described for the CARIACO Ocean Time Series program (Astor et al., 2011).

DNA collection and extraction for microbial community analyses were also described previously in Suter et al. (2018). Briefly, Niskin bottle water was sequentially filtered through a 2.7 μm glass fiber filter (EMD Millipore) in series with a 0.2 μm in-line Sterivex filter unit (EMD Millipore) which separated particle-associated (PA) from free-living (FL) microbial assemblages. Duplicate samples were collected from each size fraction at 6 depths on each date. During sample collection, Niskin bottles were slightly pressurized through their top vents with N_2_ or Ar gas in order to prevent introduction of O_2_ during water withdrawal and direct filtration. Gas pressure was enough to filter samples directly from the Niskin bottles without using a pump. After the complete Niskin bottle volume (8–12 L) passed through the filters, they were transferred immediately to a lysis buffer and stored frozen (–80°C) until further processing. DNA was later extracted from the filters as in Frias-Lopez et al. (2008) and Ganesh et al. (2014).

### Sequence Processing and Pipeline

Eukaryotic 18S rRNA genes were PCR-amplified from DNA extracts from separate filters using the TAReuk454FWD1 and TAReukREV3 primers from Stoeck et al. (2010). Four replicate PCR reactions were run for each extract and then PCR products from the same sample were pooled and purified with the Agencourt AMPure XP Kit (Beckman Coulter). Amplicons were sequenced at the Georgia Genomics Facility on Illumina MiSeq PE300. Sequences were processed by first removing primer segments with cutadapt (version 1.18; Martin, 2011) and then by implementing the DADA2 pipeline (Callahan et al., 2016) for quality filtering, merging, and ASV assignment. Taxonomic assignments were made using the reference PR2 database (version 4.12.0; Guillou et al., 2012).

### Statistical Analyses

Multivariate analyses were completed in R (version 4.0.2) using the vegan package (version 2.5-7). Principal component analysis (PCA) was performed on a center-log-ratio (clr) transformed abundance matrix of 18S rRNA ASVs. This transformation combined with PCA accounts for compositionality of these dataset types (Gloor et al., 2017). Relationships between the ordination and environmental variables or particular ASVs were determined using the vegan function envfit. Relative abundance plots of eukaryotic ASVs and PCA plots were made using the packages phyloseq (version 1.32), tidyverse (version 1.3.0), and base R functions.

The bacterial and archaeal OTU tables from contemporaneous samples reported in Suter et al. (2018) were analyzed alongside the eukaryotic ASV tables to determine potential relationships among organisms from the Bacteria, Archaea, and Eukarya domains. Tables were filtered to retain the 100 most abundant ASVs or OTUs from each domain and SpiecEasi (version 1.1.1; McMurdie and Holmes, 2013) was implemented for association inference among the 300-member dataset. SpiecEasi eliminates spurious associations that can occur in networks based on pairwise correlations like Pearson, Spearman, or SparCC (Kurtz et al., 2015). SpiecEasi determines only direct associations between members through conditional independence, which eliminates associations between taxa that may be correlated but were indirectly connected. The parameters of the spiec.easi function were carefully chosen to optimize network stability as described in the documentation^2^. We followed the approach of Tipton et al. (2018) for determining cross-domain associations. A cross-domain network was calculated using all samples (48 samples), using samples from only the oxycline (24 samples), and using samples from only the anoxic layer (16 samples). We did not have sufficient samples to derive a stable network using only the euxinic layer samples. We also repeated the network inference to determine impacts of eukaryotes on the association network: (1) by removing all eukaryotes and replacing with prokaryotes, (2) by removing eukaryotic parasitic ASVs and replacing with eukaryotic non-parasitic ASVs, and (3) by removing eukaryotic non-parasitic ASVs and replacing with eukaryotic parasitic ASVs. In all cases, the network size was retained at 300 nodes in order to control for network size, and included the 100 most abundant ASVs or OTUs from each domain after filtering. In case (1), in which all eukaryotes were removed, the top 150 bacterial and 150 archaeal OTUs were used. Parasites were identified based on literature reviews and their taxonomy is listed in Table 1.

**TABLE 1:** The eukaryotic groups annotated as parasites.

Supergroup/Division	Groups within	References
Alveolata/Dinoflagellata	Syndiniales	Guillou et al., 2008
Alveolata/Ciliophora	Oligohymenophorea	Gómez-Gutiérrez et al., 2006; Lynn, 2008
Stramenopiles/Sagenista	Labyrinthulomycetes	Raghukumar, 2002; Xie et al., 2021
Opisthokonta/Fungi	Chytridiomycota	Rasconi et al., 2011
Rhizaria/Cercozoa	Protaspa-lineage	Drebes et al., 1996; Schnepf and Kühn, 2000
Alveolata/Apicomplexa	–	Skovgaard, 2014
Alveolata/Perkinsea	–	Skovgaard, 2014

*Supergroup and division are indicated. If groups of greater taxonomic resolution within the division were considered parasites rather than the entire division, those are listed under “Groups within.” If no groups are listed in this column, then the entire division was considered as parasites.*

Networks were plotted using the iGraph package (version 1.2.6; Csardi and Nepusz, 2006), where nodes represent species and edges represent positive or negative associations. Network and node parameters were also determined using iGraph. Network parameters such as total number of edges, edge density (number of edges relative to all possible edges), components (number of “clumps” and their membership), and average path length (average shortest distance between all paired nodes) were used to determine overall network topologies. For each node, the degree (number of edges connected to the node) and betweenness centrality (the number of shortest paths going through the node) were calculated. High betweenness centrality has been used to indicate keystones that represent “bottlenecks,” or important connectors in the network, while high degree nodes indicate keystones that are “hubs” that bridge together different components (Ruiz et al., 2017; Tipton et al., 2018).

### Data and Resource Availability

All sequence data are available as a single BioProject (PRJNA326482) at NCBI. The eukaryotic 18S rRNA gene libraries correspond to sample accession numbers SRR3735256 through SRR3735305. All computational pipelines are available as open-source code at https://github.com/lizsuter/Cariaco-eukaryotes. The pipeline was run using computational infrastructure at Cyverse (Merchant et al., 2016) and the analysis environment for this project can be reproduced in the Cyverse Discovery Environment^3^ with the app “rstudio-dada2-decipher.”

## Results

### Eukaryotic Community Composition and Diversity

**FIGURE 1:**
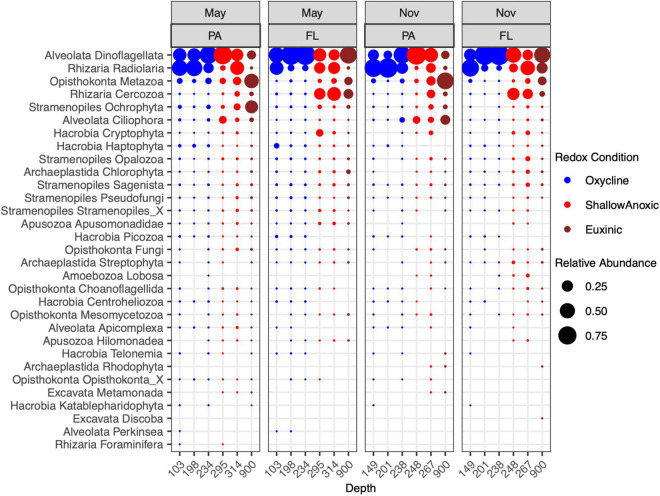
Relative abundance of 18S rRNA ASVs from May 7–9, 2014 and November 5–7, 2014 cruises in the PA (>2.7 μm) and FL (0.2–2.7 μm) fractions. Taxa were binned at the level of Phylum or Kingdom.

The most abundant 18S rRNA ASVs from this Cariaco Basin dataset were affiliated with Alveolata, Rhizaria, Opisthokonta, Stramenopiles, and Hacrobia (Figure 1). Across all samples, the most abundant division/group on average was the Dinoflagellata (51% ± 20), followed by Radiolaria (27% ± 18), Metazoa (8% ± 10), Cercozoa (7% ± 8), Ochrophyta (2% ± 5), Ciliophora (2% ± 3), and Cryptophyta (0.7% ± 1). Dinoflagellate-affiliated amplicons were abundant in both size fractions and under all redox conditions. ASVs of Radiolaria, Metazoa, Ochrophyta, and Ciliophora were more abundant in the PA fraction, while Cercozoa and Crytophyta amplicons were more abundant in the FL fraction. Metazoa, Cercozoa, Ciliophora, and Cryptophyta were also notably more abundant in anoxic and euxinic depths than in the oxycline while the others were distributed throughout all depths.

The most abundant ASVs from Alveolata were affiliated with Group II Syndiniales, followed by Group I Syndiniales (Supplementary Figure 1). Syndiniales Groups III, IV, and V were also detected. These obligate parasites were observed in samples from all depths and size fractions, but were more abundant in the FL fraction in both May and November. Relative abundances of Group II were highest in the oxycline while relative abundances of Group I were greatest at euxinic depths. The next most abundant alveolates were dinoflagellates within the Dinophyceae class: Peridiniales and a Dinophyceae group of an undetermined Order (Dinophyceae_X). These were also present at all depths and redox conditions, but most abundant in the PA fraction in shallow anoxic waters. Other abundant groups included the ciliate taxa Nassophorea, Oligohymenophorea, and Cariacotrichea. The Nassophorea were present at most depths but more abundant in the PA fraction in shallow anoxic waters while Oligohymenophorea and Cariacotrichea were only detected in shallow anoxic or euxinic waters, especially in the PA fraction in November.

Within the Rhizaria, the most abundant ASVs were identified as Spumellarida, a radiolarian from the class Polycystinea (Supplementary Figure 2). In the oxycline, these were more abundant in the PA fraction while at shallow anoxic and euxinic depths, these were more abundant in the FL fraction. Cercozoa from the class Filosa-Thecofilosea and order Cryomonadida were also abundant, particularly in the FL fraction at shallow anoxic and euxinic depths. Notably, many of the rhizarian RAD groups (RAD-A, RAD-B, RAD-C) were detected in the oxycline and shallow anoxic samples as well.

The majority of amplicons identified as Opisthokonta were affiliated with the Cnidaria, Ctenophora, and Crustacea (mainly copepods, not shown; Supplementary Figure 3). These metazoan amplicons were, expectedly, mainly found in the PA fraction. Less expectedly, their relative abundances were highest at euxinic depths. Opisthokonta ASVs were also annotated to several fungal groups, including Chytridiomycota, Cryptomycota, Basidomycota, and Ascomycota, which were detected in the May samples in both size fractions and most depths, but were mostly absent from the November samples.

The dominant class within the Stramenopiles were the diatoms (Bacillariophyta; Supplementary Figure 4), which were most abundant in the PA fraction at shallow anoxic and euxinic depths, particularly in May. The Stramenopiles were also largely composed of classes such as Chrysophyceae, Bicoecea, Labyrinthulomycetes, and the MAST groups. Many of the Stramenopile classes comprised the Sagenista, Opalozoa, and Pseudofungi phyla.

The diversity of eukaryotic communities was generally higher in the lower region of the oxycline than in samples from above or below this depth (Table 2 and Supplementary Figure 5). Profiles of Shannon’s Diversity Index (*H’*) for both cruise dates and size fractions revealed that diversity usually declined from the oxycline into the shallowest anoxic sample, then increased again in the deeper anoxic sample. The May FL fraction was an exception where *H’* only declined from the oxycline through the anoxic layer. Notably, Shannon’s diversity index was similar in the euxinic layer and in the oxycline in November samples.

**TABLE 2:** Shannon’s diversity index (*H’*) based on eukaryotic ASVs for each cruise, depth, and size fraction (PA, particle-associated; FL, free-living).

Layer	May depth [m]	*H’:* May PA	*H’:* May FL	November depth [m]	*H’:* Nov. PA	*H’:* Nov. FL
Oxycline	103	4.9 (0.1)	4.6 (0.6)	143	3.9 (1.1)	4.0 (1.4)
	198	4.4 (0.0)	5.2 (0.1)	200	3.1 (0.0)	4.8 (0.0)
	234	5.0 (0.5)	5.1 (0.1)	237	4.3 (0.1)	2.9 (0.7)
Anoxic	295	2.9 (0.3)	3.1 (0.6)	247	2.4 (0.0)	2.7 (0.1)
	314	4.5 (0.0)	3.0 (0.3)	267	3.9 (0.1)	4.4 (0.0)
Euxinic	900	3.3 (0.5)	2.6 (0.7)	900	3.0 (0.0)	4.5 (0.0)

*Standard deviations are derived from duplicate samples and listed in parentheses.*

### Multivariate Analyses

**FIGURE 2:**
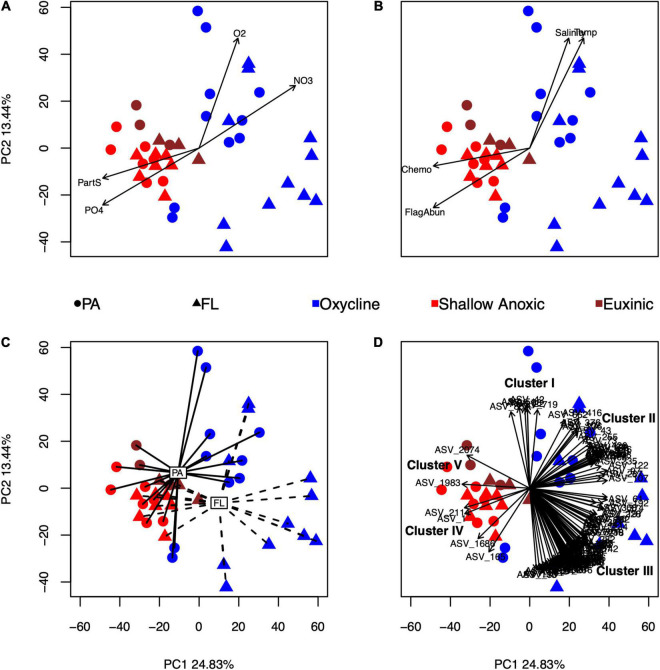
Principal component analysis (PCA) of the eukaryotic amplicon ASV tables after a CLR transformation. The first and second components describe 24.83 and 13.44% of the variance, respectively. Each symbol represents an individual sample, color-coded by redox condition and shape-coded by size fraction. Environmental variables that were significantly correlated with the ordination using envfit are shown as vectors or centroids: oxygen (O_2_), nitrate (NO_3_^–^), particulate sulfur (PartS), and phosphate (PO_4_^3–^) in panel **(A)**; salinity (Salinity), temperature (Temp), microscopic cell counts of flagellated protists (FlagAbun), and rates of chemoautotrophic production (Chemo) in panel **(B)**; and size fraction (PA, particle-associated; FL, free-living) in panel **(C)**. Individual ASVs that were significantly correlated to the ordination (*p* < 0.001, *r*^2^ > 0.60) are shown in panel **(D)**. Covarying ASVs were divided into five clusters (I–V), further described in the text.

The first two components of the principal components analysis (PCA) of the eukaryotic ASV tables explained a total of 38.3% of variability in eukaryotic community composition (Figure 2). Several environmental variables significantly correlated with the composition of 18S rRNA amplicons: oxygen, nitrate, particulate sulfur, phosphate, temperature, salinity, flagellate cell counts, rates of chemoautotrophic production, and size fraction (Figures 2A–C). Furthermore, a subset of 107 ASVs were found to be highly correlated with the ordination at the *p* < 0.001 and *r*^2^ > 0.60 level (Figure 2D).

Based on the directions of the relationships of the 107 correlated ASVs in the PCA (Figure 2D and Supplementary Table 1), the ASVs were split into five clusters. Cluster I consisted of four Spumellarida in the Spongodiscidae-Coccodiscidae family and two Group II Syndiniales. Based on their position, ASVs from this cluster were affiliated with higher oxygen, salinity, and temperature, and with samples from the PA oxycline fraction. Cluster II, which consisted of 24 ASVs, was mainly composed of Group II Syndiniales. Additionally, there was one radiolarian (RAD-B), one Picozoa (supergroup: Hacrobia), and six Group I Syndiniales in this cluster. The position of this cluster in the ordination suggested these ASVs were associated with higher nitrate values. Cluster III, which contained 71 ASVs, was also mainly composed of Group II Syndiniales. Additionally, there were two Dinophyceae ASVs in this cluster with ambiguous assignments at lower taxonomic levels and two Group V Syndiniales. Based on their position, ASVs in this cluster were associated with the FL oxycline samples. Cluster IV contained four ASVs; one Bacillariophyta (diatom), one Gymnodinium (dinoflagellate), and two Spumellarida. Their position in the ordination indicates ASVs from this cluster were affiliated with anoxic samples and high phosphate and particulate sulfur. Lastly, Cluster V, which contained only two Bacillariophyta ASVs, was affiliated with the euxinic samples.

### Network Analyses

**FIGURE 3:**
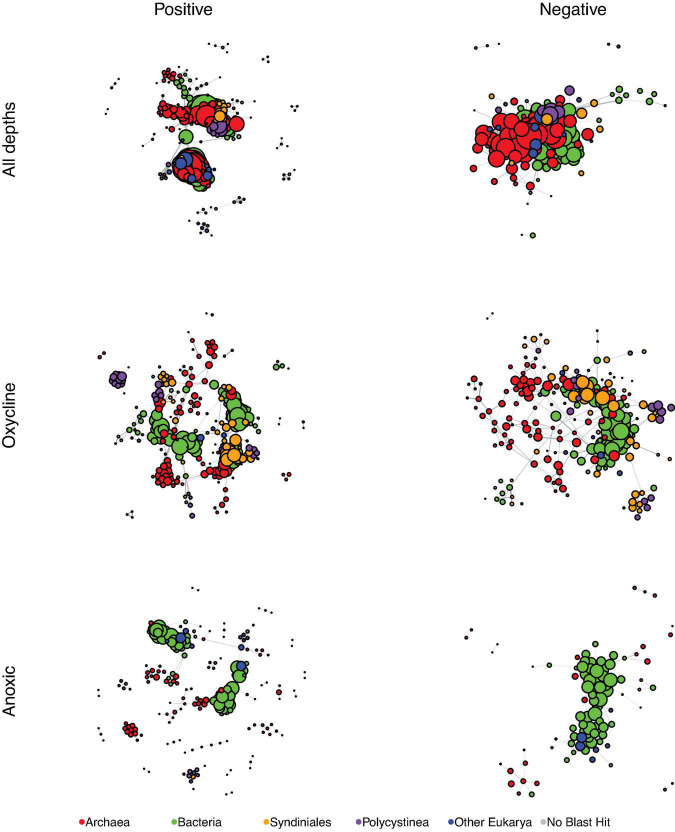
SpiecEasi-based networks of the combined eukaryotic ASV and prokaryotic OTU tables. “All depths” indicates the network calculated from the entire dataset (oxycline, anoxic, and euxinic samples with taxonomic information from both the prokaryotic and eukaryotic tables). “Oxycline” is a network using samples only from oxycline depths. “Anoxic” is a network using samples only from shallow anoxic depths. After filtering, any node that was no longer connected to another node was removed from the graph. Node size is reflective of the number of edges that are connected to that node. Each network was filtered to present only positive edges **(left)** and only negative edges **(right)**. Nodes are color-coded to indicate Bacteria, Archaea, the eukaryotic groups Syndiniales and Polycystinea, and all other Eukarya. Every network was controlled for size and contains 300 members.

**FIGURE 4:**
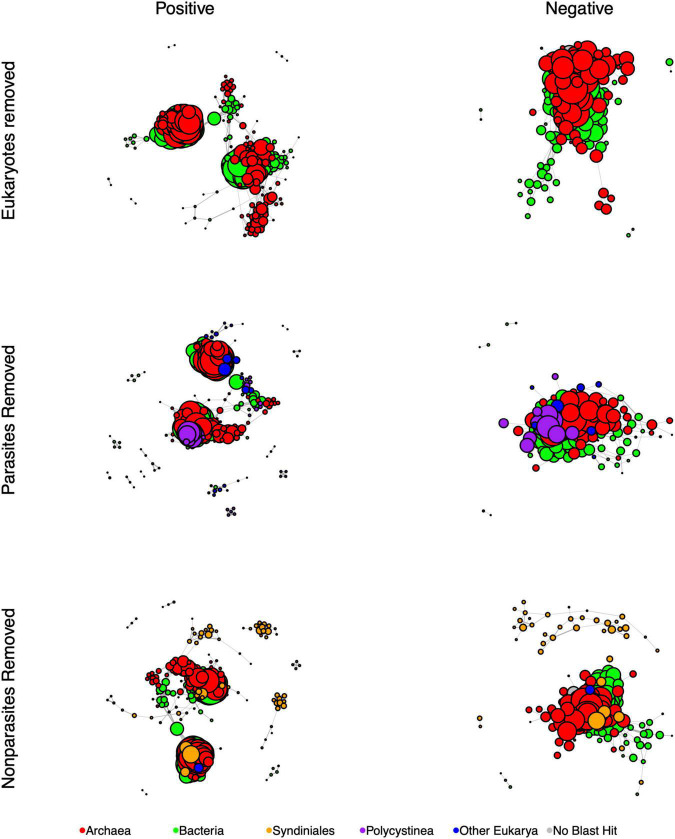
SpiecEasi-based networks of the combined eukaryotic ASV and prokaryotic OTU tables after filtering in order to determine the impact of eukaryotes (compare to “All depths, Figure 3). “Eukaryotes removed” indicates the network calculated after removing all eukaryotic ASVs. “Parasites removed” is a network after removing eukaryotic parasites (listed in Table 1). “Non-parasites removed” is a network that includes only parasitic eukaryotes. After filtering, any node that was no longer connected to another node was removed from the graph. Node size is reflective of the number of edges that are connected to that node. Each network was filtered to present only positive edges **(left)** and only negative edges **(right)**. Nodes are color-coded to indicate Bacteria, Archaea, the eukaryotic groups Syndiniales and Polycystinea, and all other Eukarya. Every network was controlled for size and contains 300 members.

To determine potential relationships between eukaryotes and prokaryotes, SpiecEasi association matrices were built from eukaryotic ASV and prokaryotic OTU tables, and the matrices were used as input for building networks. Each network is presented as a positive associations graph and negative associations graph (Figures 3, 4), where all associations are represented by lines (“edges”) between individual ASVs or OTUs (“nodes”). While they were not the most connected nodes, ASVs from the Syndiniales and Polycystinea taxa were among the most abundant eukaryotic nodes in the full-dataset network (Figure 3), and were better represented among the positive associations than among the negative associations. Syndiniales represented 26 nodes from the positive association network (1 from Group I and 25 from Group II) and 9 from the negative association network (1 from Group I and 8 from Group II). The polycystine order, Spumellarida, was represented by 28 nodes in the positive association network and 7 nodes in the negative network.

We further examined which nodes were the nearest neighbors of Syndiniales and Spumellarida nodes. In the positive association networks, Syndiniales were most commonly connected to other Syndiniales, Spumellarida, bacteria in the Marine group A taxon (Marinimicrobia; formerly SAR406) and euryarchaeota in the Marine Group II clade while Spumellarida were most commonly associated with Syndiniales (Group II), and members of the euryarchaeota Marine Group II clade. In the negative association networks, Syndiniales were most connected to bacteria in Candidate division WS3, while Spumellarida were most connected to Marine Group A.

A ciliate ASV from the class Cariacotrichea, first identified in the Cariaco Basin, was also present in the network. Because this ciliate was previously shown by SEM to contain unidentified epibionts, we also investigated the positive associations between Cariacotrichea and prokaryotes in the network. Cariacotrichea was positively associated with a planctomycete ASV (MSBL9 in the Phycisphaerae), two deltaproteobacteria (Desulfarculales and Sh765B-TzT-29), two spirochetes (MSBL8), and a Candidate division OP3 OTU (*candidate phylum* Omnitrophica; Rinke et al., 2013).

Individual SpiecEasi association matrices and networks were also built by subsampling from only the oxycline or only shallow anoxic depths (Figure 3). Syndiniales and Polycystinea nodes were also abundant in the oxycline networks, where they represented a total of 81 nodes in the positive association network, and 58 in the negative association network. Syndiniales were not a major component of the anoxic network; seven Group II nodes were present among the positive associations and one Group I node among the negative associations. Spumellarida nodes were abundant in the positive association anoxic network (15 nodes) and more rare (2 nodes) in the negative association network from these anoxic depths.

Network topological features were also calculated for the complete set of associations (positive and negative) for each graph, while controlling for network size (Table 3). The oxycline and anoxic networks differed from the full network. The total number of edges and edge density were highest for the full network and lowest for the anoxic network. The oxycline network exhibited the highest proportion of negative associations. The oxycline network also had the fewest number of components (“clumps”) and the largest sized largest component of the three. The anoxic network contained the most components, and smallest sized largest component of the three. Filtering the entire dataset to just oxycline samples or just anoxic samples increased the average path length from 3.1 to 4.1 and to 5.3 for the oxycline and anoxic networks respectively. Overall, these parameters suggested that the oxycline network was more highly connected than the anoxic network, with shorter distances between nodes, while the anoxic network was more disjointed, with smaller, more distantly connected hubs.

**TABLE 3:** Network parameters of the SpiecEasi-based networks calculated from the ASV and OTU tables.

	Number of edges	Edge density	% Negative edges	Number of components (“Clumps”)	Size of largest component	Average path length
All depths; all domains	2468	5.5	33.6	38	233	3.1
Oxycline; all domains	1535	3.4	39.2	23	274	4.1
Anoxic; all domains	767	1.7	32.9	113	140	5.3
All depths; all eukaryotes removed	3450	7.7	30.8	21	276	3.3
All depths; parasitic eukaryotes removed	2824	6.3	35.5	41	222	2.8
All depths; non-parasitic eukaryotes removed	2680	6.0	37.8	40	257	4.6

*A network was derived utilizing all samples from the study (All depths; all domains), based on only oxycline samples (Oxycline; all domains) and only shallow anoxic samples (Anoxic; all domains). The full network (all depths) was also then recalculated after removing eukaryotic AVSs (All depths; eukaryotes removed), after removing eukaryote ASVs annotated as parasites (All depths; parasitic eukaryotes removed) and after removing eukaryote ASVs that were non-parasites (All depths; non-parasitic eukaryotes removed). Before calculating networks, all datasets were filtered to included only the top 300 most abundant nodes. For most networks, the top 100 bacteria, archaea, and eukarya were included. For the prokaryote-only network (All depths; eukaryotes removed), the top 150 bacteria and archaea were chosen.*

To test for the impact of eukaryotes and specifically eukaryotic parasites on the overall network, we also re-calculated network topological features after removing-and-replacing all eukaryotic ASVs with prokaryotes, removing-and-replacing all parasitic eukaryote ASVs with non-parasites, or by removing-and-replacing all non-parasitic eukaryote ASVs with parasites while controlling for network size (Table 3 and Figure 4). ASVs functionally annotated as parasites (Table 1) made up 45% of the entire eukaryotic 18S rRNA ASV database. Removing and replacing all eukaryotes had the greatest impact, by greatly increasing the number of edges, edge density, and size of largest component while decreasing the percentage of negative edges and total number of clumps compared to the full dataset, suggesting that the lower abundance prokaryote nodes that replaced the eukaryotes in the network had higher overall degrees and fewer negative associations than eukaryotes. Both replacing parasites and replacing non-parasitic eukaryotes in the network also increased the number of edges and edge density, while increasing the number of clumps. This suggests that, in the overall network, associations *between* eukaryotic parasites and non-parasites had maintained a lower number of clumps with fewer edges between them. Average path length also decreased in the absence of parasites but increased when non-parasites were replaced. Thus, the impact of parasites specifically was to increase the average distances between nodes.

We also determined potential keystone taxa from the full dataset based on betweenness centrality and node degree, according to Ruiz et al. (2017) and Tipton et al. (2018). Nodes with the highest betweenness centrality, which represent likely bottlenecks, included OTUs from Marine Group I (Thaumarchaeota) and Marine Group A (Deferribacterales), while nodes with the highest degree, which represent likely hubs, included Alphaprotebacteria (SAR11) OTUs, and an MSBL8 OTU (Spirochaetes; Supplementary Figure 6). Nodes with both high degree and high betweenness included an OTU from SAR11 Deep-1 clade and an Acidimicrobiales OTU (Actinobacteria). While no eukaryotes were identified as potential keystone taxa, Syndiniales (majority of Dinoflagellata nodes; yellow squares in Supplementary Figure 6) and Spumellarida (majority of Radiolaria nodes; red squares) were abundant nodes in the network and several Syndiniales nodes had relatively high betweenness measures, indicating a possible role as network bottlenecks. As an aggregate, Candidate division WS3 (“uncultured_bacterium” in Supplementary Figure 6) and Marine Group II (Thaumarchaeota) represented the highest number of edges in the network, followed by Radiolaria (the majority of which were Spumellarida), Alphaprotebacteria (the majority of which were SAR11), and Dinoflagellata (the majority of which were Group II Syndiniales).

## Discussion

### Eukaryotic Community Composition

Representatives of the eukaryotic taxonomic group, Syndiniales, were consistently important throughout this analysis in terms of ASV relative abundance, correlations in the multivariate analysis, and connectedness in networks. Syndiniales are widespread marine protists that have been reported commonly in 18S rRNA gene libraries from the open-ocean (Guillou et al., 2008; de Vargas et al., 2015), coastal ecosystems (Park et al., 2004; Guillou et al., 2008; Anderson and Harvey, 2020; Rocke et al., 2020), and polar systems (Cleary and Durbin, 2016; Clarke et al., 2019). They are particularly abundant in ODWCs (Parris et al., 2014; Duret et al., 2015; Torres-Beltrán et al., 2018; De la Iglesia et al., 2020). Despite being found throughout the modern-day ocean, genetic signatures from the fossil record also suggest that Syndiniales are strong indicators of oxygen-depleted conditions (More et al., 2018).

Syndiniales are taxonomically divided into groups I–V and while all described species are obligate parasites, some species or strains may have non-specific hosts (Guillou et al., 2008). Group II, which was the most abundant group in this study, is a genetically diverse group with several dozen clades associated with both aphotic and euphotic planktonic ecosystems while Group I, which has fewer clades, is more often associated with low-oxygen waters (Guillou et al., 2008; Duret et al., 2015). In this study, Group I ASVs were more abundant at anoxic depths (O_2_ = 0 and presence of H_2_S) while Group II was found mainly in the oxycline. Similar to our study, Syndinales have previously been reported both in FL and PA fractions in ODWCs (Parris et al., 2014; Duret et al., 2015), which is thought to reflect their parasitic life cycles; small spores are released following infection events and detected in the FL fraction while active host infections are detected in the PA fraction (Guillou et al., 2008).

Polycystine radiolaria in the order Spumellarida were also very abundant in this study at all depths and in both size fractions. Polycystine radiolaria have been reported previously in ODWCs (Orsi et al., 2011; Parris et al., 2014) and also in the fossil record during periods of intensified oxygen depletion (More et al., 2018). Most described polycystines have endosymbiotic algae, which can be dinoflagellates, prasinophytes, or prymnesiophytes (Anderson, 1983; Takahashi et al., 2003). Polycystines have frequently been reported to co-occur with, and to be potentially parasitized by Syndiniales (Not et al., 2007; Guillou et al., 2008; Xu et al., 2018). On a global scale, recoveries of both Syndiniales and polycystine amplicons are relatively high in ODWCs.

The relative abundances of Syndiniales and polycsytines may be overestimated compared to their actual cell number and activity (Giner et al., 2020). As is the case with other protist taxa, dinoflagellates often have high numbers of SSU rRNA gene copies (Galluzzi et al., 2010; Lin, 2011), and Syndiniales release numerous spores, which may overestimate their relative abundances. Siano et al. (2011) reported that Syndiniales dinospores comprised up to 35% of the eukaryotic community in the Mediterranean Sea determined by microscopy. It is also possible that the ASV approach, which is sensitive to intragenomic variation, can overestimate the abundances of species with high numbers of SSU rRNA gene copies (Schloss, 2021). However, Syndiniales signatures are consistently one of the most abundant, if not the most abundant eukaryotic taxa in studies that perform 97% OTU clustering (e.g., de Vargas et al., 2015; Pernice et al., 2016; Clarke et al., 2019). Additionally, the most abundant bacterial ASV in this study, a chemoautotrophic gammaproteobacterium (Suter et al., 2018), was *not* highly connected in the network analysis, suggesting that high abundance alone does not drive connectivity of a node in network analysis. Furthermore, while individual associations between nodes are driven by co-occurring abundance patterns that could be impacted by over- or underestimating species abundances from ASV hits, this would not impact the interpretation when comparing the different ASV-based networks to each other. Thus, the network topology conclusions based on removing groups of eukaryotes, parasites, and non-parasites also imply that Syndiniales and Spumellarida have important impacts within this community.

Other abundant genetic signatures in the anoxic and euxinic layers examined in this study were members of the Cercozoa (class Filosa-Thecofilosea; in the order Cryomonadida), Stramenopiles within Chrysophyceae, Sagenista, Opalozoa, and Pseudofungi, and RAD groups within the Radiolaria (RAD A, B, and C). Thecofilosea (within Cercozoa) have previously been shown to be indicators of oxygen-depleted conditions in the sediment record (More et al., 2018). In this study, these amplicons were found in highest relative abundances in the deepest, euxinic samples collected at 900 m. Similarly, many of the Stramenopiles detected in this study have been described previously from habitats with oxygen-sulfide gradients (Behnke et al., 2010), including earlier clone libraries from the Cariaco Basin (Orsi et al., 2011), and were shown to be enriched in anoxic systems compared to other aquatic ecosystems by a meta-analysis (Massana et al., 2014). Cercozoa have been shown to be enriched in the bathypelagic ocean (2000–4000 m; Xu et al., 2018) and have higher relative activities at these depths than in the photic zone or mesopelagic layers (Giner et al., 2020). Similarly, heterotrophic RAD groups such as RAD-B have also been shown to be enriched in bathypelagic layers (Giner et al., 2020).

Several amplicons detected at depth (900 m; euxinic) were somewhat unexpected. Strictly phototrophic Bacillariophyta (diatoms) were enriched at 900 m, particularly in May in the PA fraction, and affiliated with euxinic samples in the multivariate analyses. Upwelling at this station occurs seasonally January to May, suggesting that this diatom signature is a result of ballasted sinking particles from the spring bloom. Genetic signatures from diatoms have previously been detected in high relative abundances at depths of up to 4,000 m (Xu et al., 2017), have been recovered from deep-sea sediments (Orsi et al., 2013), and even live diatom cells have been detected as deep as 2,000–4,000 m at various stations throughout the world ocean (Agusti et al., 2015), suggesting that these particles are delivered to depth in a matter of days. Metazoan sequences were also abundant at 900 m, particularly in the PA fraction but also in the FL fraction. Metazoa are likely overrepresented relative to protists in amplicon surveys due to their multicellularity. However, cnidarians and ctenophores have been reported in oxyclines of some of the ocean’s major oxygen-depleted zones (Purcell et al., 2001). In the Black Sea, for example, ctenophores have been observed in the upper anoxic layers in the presence of sulfide (Mutlu and Bingel, 1999). Metazoa such as copepods and small mesopelagic fishes may migrate temporarily into anoxic layers to feed on abundant microbial life along lower oxyclines where there is less competition for prey or to escape predators in shallower waters (Baird et al., 1974; Wishner et al., 2008, 2013).

### Parasitism

Parasitism is relatively understudied in marine systems compared to terrestrial systems (Käse et al., 2021). The focus of many marine microeukaryotic community studies has largely been predator-prey relationships even though parasitic relationships are thought to comprise a large proportion of interactions in communities. In a review of the protistan interactome, predation was the most common interaction, but parasitism comprised at least 18% of interactions (Bjorbækmo et al., 2020). In a recent study of North Sea surface waters, 18S rRNA gene metabarcoding revealed that parasites contributed at least 10% of OTUs, which is likely an underestimate because trophic modes of 55% of total OTUs were unresolved (Käse et al., 2021). In Saanich Inlet, an anoxic fjord, Syndiniales parasites made up more than 40% of 18S rRNA OTUs in select samples (Torres-Beltrán et al., 2018). Similarly, in the current study, eukaryotic ASVs annotated as parasites made up 45% of the entire eukaryotic ASV library, suggesting that parasites could play an outsized role in oxygen-depleted marine systems. The free-living spore stages of parasites, such as Syndiniales, can spread widely in marine environments by diffusion and circulation and are not subject to desiccation stress as they would be in terrestrial environments (de Vargas et al., 2015), making parasitism an advantageous strategy in marine systems. Furthermore, the impact of parasites in the food web may be even greater than their numerical abundances suggest, particularly for host-promiscuous parasites that can interact with a large variety of other eukaryotes (Hudson et al., 2006).

Generally, Syndiniales have been reported to parasitize many other eukaryotes, including both protists and metazoa. One described genus from Group II, *Amoebophyra*, which contains at least 7 described species, has been shown to parasitize dinoflagellates, including other Syndiniales, radiolarians, ciliates, and metazoa such as Cnidaria (Jephcott et al., 2016). The *Amoebophyra* comprise only a small fraction of the genetic diversity of Group II (Guillou et al., 2008) and thus the potential hosts of Group II are likely very broad. Described species from Group I include members that parasitize fish eggs and ciliates (Guillou et al., 2008). Clone libraries from radiolarian isolates revealed that Group I Syndiniales may be parasites of polycystine radiolaria, or parasites of their symbionts (Dolven et al., 2007; Guillou et al., 2008). Studies in the Southern Ocean also revealed correlations between Syndiniales parasites and radiolarian groups (Cleary and Durbin, 2016). Thus, consistent with results from this study, hosts of Syndiniales likely include radiolaria, such as Spumellarida, as well as other dinoflagellates.

Other ASVs in this dataset potentially arising from parasitic taxa included the Oligohymenophorea (ciliates), Labyrinthulomycetes (Stramenopiles), and chytrids (fungi). Species within the Oligohymenophorea were shown to be parasites of metazoa such as euphasiids (Gómez-Gutiérrez et al., 2006) and have been found in association with ctenophores (Estes et al., 1997) and copepods (Skovgaard, 2014). Members of the Labyrinthulomycetes can be parasites, bacterivores, or saprophytes (Xie et al., 2021). Species of fungal chytrids are diverse parasites of fish, eggs, zooplankton, and phytoplankton (Rasconi et al., 2011). Further studies with ITS primers would be needed to determine the full diversity of fungi at this site. The abundant Cryomonadida (Cercozoa; Filosa-Thecofilosa) sequences were either annotated to *Protaspa* or were unassignable at higher taxonomic resolution. Members of the Protaspa have been shown to feed on diatom cytoplasm with pseudopodia that penetrate the host shell (Drebes et al., 1996; Schnepf and Kühn, 2000). Furthermore, we detected ASVs within the Apicomplexa and Perkinsida (alveolates) at low relative abundances. These taxa contain known parasites that infect other protists and invertebrates (Skovgaard, 2014).

While the role of parasites in marine microbial food webs is not well described, limited work from other ecosystems and cultures suggests that they are pivotal in structuring community species composition and altering productivity (Hatcher et al., 2012). Parasitic infections regulate host abundances, in turn shaping host population structure (Rasconi et al., 2011). For example, parasites have been shown to terminate algal blooms in surface waters (Chambouvet et al., 2008). The impact of parasitic infection on host population abundance can be of the same order of magnitude as predation (Skovgaard, 2014). In terrestrial systems, active recurring parasitic infections have been shown to drive diversification of host populations (Janz et al., 2006). Non-lethal parasites can decrease host fitness, which changes host populations by compromising reproductive output (Skovgaard, 2014). Large-scale infections can also transfer carbon in ecosystems where energy may be in short supply, such as oxygen-depleted water columns. Rapid production of spores that are similar in abundance and size to prey items can transfer energy to higher trophic levels through grazing (Rasconi et al., 2011). Furthermore, while about half of the host biomass is converted to spores during an infection, the other half is assumed to contribute to the dissolved and particulate organic matter (DOM, POM) pools (Jephcott et al., 2016). Thus, during large-scale infections in ODWCs, parasites likely affect food webs in significant ways (Torres-Beltrán et al., 2018).

### Impacts of Parasites on Overall Ecosystem Structure

We tested the impacts of eukaryotes and parasitic eukaryotes on the association networks by successively removing and replacing groups and recalculating network topologies. Including eukaryotes led to less densely linked networks, with more clumps and shorter path lengths, while including parasites increased the path lengths between nodes. In a similar study of the human lung and skin microbiome, including eukaryotic fungi with bacterial and archaeal domains resulted in a more connected and stabler network with shorter path lengths (Tipton et al., 2018), however those fungi were not necessarily parasites. Few other studies have attempted to determine interactions among marine microbial community members from all three domains. However, beyond microbial food webs, the impacts of including parasites in food web network models has suggested that they increase link density and food chain length (Lafferty et al., 2006a; Dunne et al., 2013). For example, in a salt marsh study, parasites increased the number of links and path lengths in the food web, which increased ecosystem stability and resilience (Hudson et al., 2006; Lafferty et al., 2006b). Inclusion of parasites in a subarctic lake food web model increased the length of food chains and overall number of links (Amundsen et al., 2009). Thus, parasites are likely increasing the number of steps in the Cariaco Basin microbial networks and maintaining high complexity, particularly in the oxycline where their relative abundances are highest.

Oxygen-depleted water columns are known to be hotspots of protist diversity (Orsi et al., 2012c). In the current study, depth profiles of eukaryotic diversity (*H’*) generally showed a decrease in diversity from the oxycline to deeper depths, mirroring diversity profiles of bacteria (Suter et al., 2018) with one major exception. In most of the depth profiles of *H’*, eukaryotic diversity exhibited a localized maximum in lower anoxic layers before declining again. For Bacteria and Archaea, diversity remained low in this layer. However, this layer is where we consistently observe localized peaks in both heterotrophic and chemoautotrophic bacterial production in contemporaneous samples (Tuttle and Jannasch, 1979; Taylor et al., 2001; Scranton et al., 2020). Such opposing relationships between bacterial diversity and bacterial productivity are well described. For example, in microbial mats, a low diversity of a few prokaryotic specialists dominates regions of high primary productivity (Bernstein et al., 2017). In the Cariaco Basin, the link between high chemoautotrophic and heterotrophic productivity, low prokaryotic diversity, and high eukaryotic diversity may partly result from interactions driven by protists. A high diversity of biotic interactions, such as predation and mutualism, can help to release or share substrates that would stimulate high rates of prokaryotic activity driven by a few prokaryotic specialists.

In network analyses, positive associations between parasites and other organisms are viewed as potential parasitic interactions (Berdjeb et al., 2018). However, positive associations between parasites and heterotrophic bacteria may also indicate that cell lysis from eukaryotic parasitism or predation leads to the release of dissolved organic matter, which in turn drives bacterial activity, similar to the viral shunt (Jephcott et al., 2016). We observed that Syndiniales were linked to a large proportion of positive associations in the networks, and were involved in specific associations with bacteria from Marine group A (Marinimicrobia) and euryarchaeota from the Marine Group II clade. Each of these prokaryotic groups is described only through their ecological distributions or metagenome-assembled genomes (MAGs) and lack cultured representatives, making their functional characterization challenging. Marine group A is associated with low-oxygen to anoxic environments (Bertagnolli et al., 2017) and iTAGs of both groups were recovered previously in abundance from the Cariaco Basin (Suter et al., 2018). Evidence suggests that members from these groups potentially remineralize carbon compounds through either aerobic or anaerobic respiration, but further analyses are needed to confirm their exact functional roles (Zhang et al., 2015; Bertagnolli et al., 2017).

### Mutualism

Modeling studies suggest that anoxic conditions select for mutualism and enhanced cross-feeding among microbial community members (Heinken and Thiele, 2015). This is a possible explanation for the differences in network topological features observed among geochemical regimes in the current study. Compared to the oxycline network, the anoxic network contained a higher number of tightly clustered clumps, with less connectedness between clumps. While oxycline samples were dominated by Syndiniales groups, anoxic and euxinic samples contained a high diversity of other eukaryotes, including ciliates. Energy-limited marine environments, such as anoxic water columns and sediments, are inhabited by diverse ciliates that host symbiotic bacteria and archaea (Orsi et al., 2012a; Edgcomb and Pachiadaki, 2014). For example, a ciliate collected from both Mediterranean and Caribbean Sea sediments was shown to host thioautotrophic gammaprotebacteria (Seah et al., 2017). Furthermore, a ciliate from anoxic-sulfidic sediments of the Santa Barbara Basin hosted at least four different endobionts that were potentially capable of sulfate reduction, methane oxidation, methanogenesis, and generation of hydrogen and acetate (Edgcomb et al., 2011c). Relationships such as these are likely mutualistic, as the symbionts rely on host metabolites, the host benefits from the production and drawdown of substrates by its symbionts, and the symbionts also live in an homeostatic environment largely protected from predators (Edgcomb et al., 2011c; Edgcomb and Pachiadaki, 2014).

The Cariaco Basin has previously been shown to host diverse ciliates (Edgcomb et al., 2011a), and over 90% of those found in anoxic samples were microscopically observed to host epibionts (Edgcomb et al., 2011b). Similar to the current study, marker gene analyses suggested Oligohymenphorea and Cariacotrichea are commonly found along marine oxyclines (Edgcomb and Pachiadaki, 2014). Cariacotrichea is a novel class of ciliate that was first identified from the Cariaco Basin and observed to have rod-shaped epibionts (Stoeck et al., 2003; Orsi et al., 2012b), the identity of which are unknown. Based on network analysis in the current study, ASVs from class Cariacotrichea were positively associated with several bacterial OTUs, including Phycisphaerae, Deltaproteobacteria (Desulfarculales and Sh765B-TzT-29), spirochetes, and Candidate Division OP3. The Desulfarculales are a particularly interesting candidate as a potential symbiont for Cariacotrichea. These are a sulfate-reducing clade, members of which have been shown to carry genes for acetogenesis and fermentation (Jochum et al., 2018). Confirmation of such a relationship would have to be made by other methods, such as microscopy and FISH.

## Conclusion

Oxygen-depleted water columns like the Cariaco Basin have previously been recognized as hotspots of microbial activity (Taylor et al., 2001) and protistan diversification (Orsi et al., 2011). The Cariaco Basin’s anoxic layer consistently exhibits high rates of chemoautotrophic and heterotrophic activity that, at times, matches and even exceeds rates of primary productivity observed in surface waters (Tuttle and Jannasch, 1979; Taylor et al., 2001; Scranton et al., 2020). Furthermore, organic matter exported to 400m is labile, chemically different, and occasionally greater in quantity than organic matter exported into traps at shallower depths (Thunell et al., 2000; Taylor et al., 2001; Li et al., 2012), suggesting that microbial communities significantly rework organic matter sinking past this midwater zone. This reworking is driven by a productive microbial food web that includes a diverse set of protists that likely mediate a large number of biotic interactions, including predation, symbiosis, and parasitism. While ecological interactions, such as predation and symbioses, have been recognized previously as important in ODWCs, parasitism has thus far been largely underappreciated. In addition to direct impacts on host populations, parasites can have indirect impacts on microbial community members by releasing organic matter during infection events, which in turn stimulates prokaryotic activity. Network analysis allowed for guided hypothesis generation about specific interactions between partners, such as Syndiniales and prokaryotic heterotrophs, or symbioses between Cariacotrichea and certain prokaryotic groups. These should be further tested by independent methods and more intensive or a higher resolution sampling scheme. Network analyses also revealed that inclusion of parasites led to a more complex network with longer paths between nodes. Thus, protists, and especially abundant protistan parasites, in the Cariaco Basin’s oxygen-impoverished layers likely have a the role of maintaining trophic complexity of the overall food web structure by providing new sources of labile organic matter along the redoxcline.

## Data Availability Statement

The datasets presented in this study can be found in online repositories. The names of the repository/repositories and accession number(s) can be found below: https://www.ncbi.nlm.nih.gov/, Bioproject # PRJNA326482.

## Author Contributions

ES, MP, GT, and VE conceived the study, the sampling schemes, and participated in fieldwork. ES and MP performed the laboratory analyses. ES performed the bioinformatics and statistical analyses. ES wrote the manuscript with advice from VE and significant input from MP and GT. All authors contributed to the article and approved the submitted version.

## Conflict of Interest

The authors declare that the research was conducted in the absence of any commercial or financial relationships that could be construed as a potential conflict of interest.

## Publisher’s Note

All claims expressed in this article are solely those of the authors and do not necessarily represent those of their affiliated organizations, or those of the publisher, the editors and the reviewers. Any product that may be evaluated in this article, or claim that may be made by its manufacturer, is not guaranteed or endorsed by the publisher.

## Footnotes

1
http://www.imars.usf.edu/cariaco

2
https://github.com/zdk123/SpiecEasi

3
https://de.cyverse.org/
