# Supplementary material for: Eukaryotic Parasites Are Integral to a Productive Microbial Food Web in Oxygen-Depleted Waters
Source: Front Microbiol. 2022 Jan 6;12:764605. doi: 10.3389/fmicb.2021.764605 (PMC8770914; doi:10.3389/fmicb.2021.764605)
Supplement: Supplementary file 1 [file Data_Sheet_1.pdf]

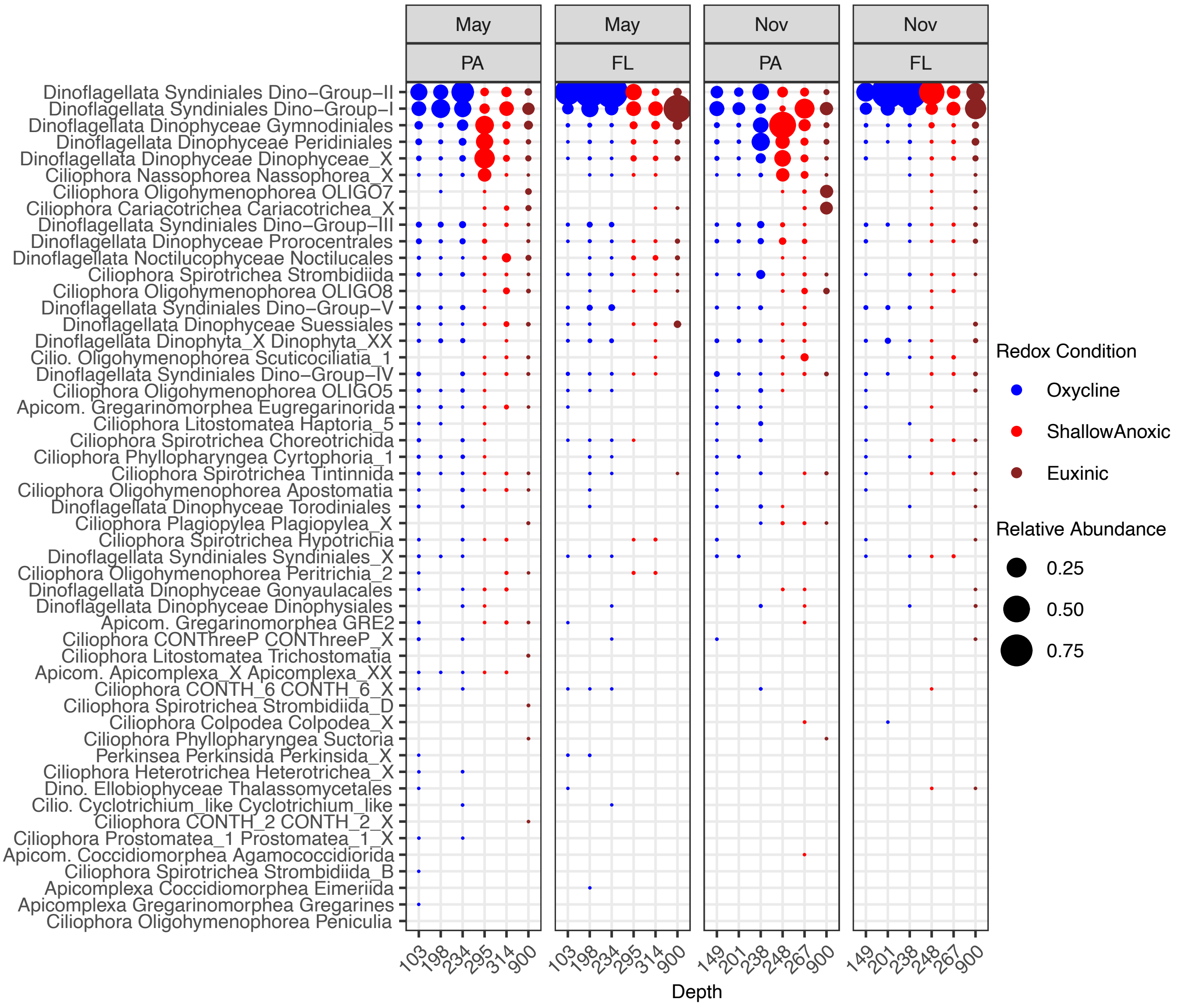

Figure S1: Relative abundance of 18S rRNA ASVs within the Alveolata superphylum from May 7-9, 2014 and November 5-7, 2014 cruises and both size fractions (PA and FL). Taxa were binned at the level of Order. Some taxa names were shortened to save space: Dino = Dinoflagellata; Cilio = Ciliophora; Apicom = Apicomplexa.

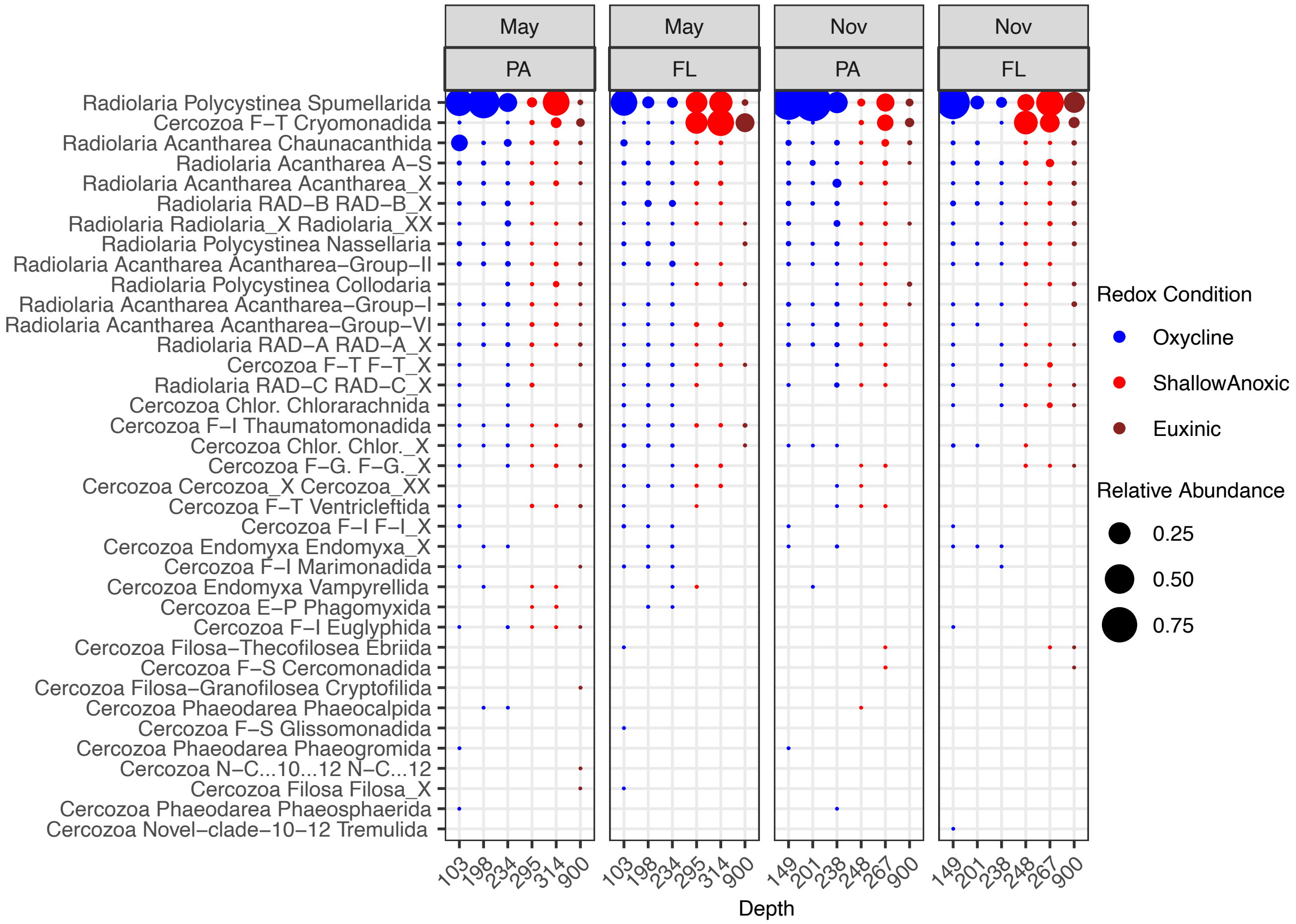

Figure S2: Relative abundance of 18S rRNA ASVs within the Rhizaria supergroup from May 7-9, 2014 and November 5-7, 2014 cruises and both size fractions (PA and FL). Taxa were binned at the level of Order. Some taxa names were shortened to save space: F-T = Filosa-Thecofilosea; A-S = Arthracanthida-Symphyacanthida; Chlor = Chlorarachniophyceae; F-G = Filosa-Granofilosea; F-I = Filosa−Imbricatea; E-P = Endomyxa−Phytomyxea; F-S = Filosa−Sarcomonadea; N-C = Novel−clade.

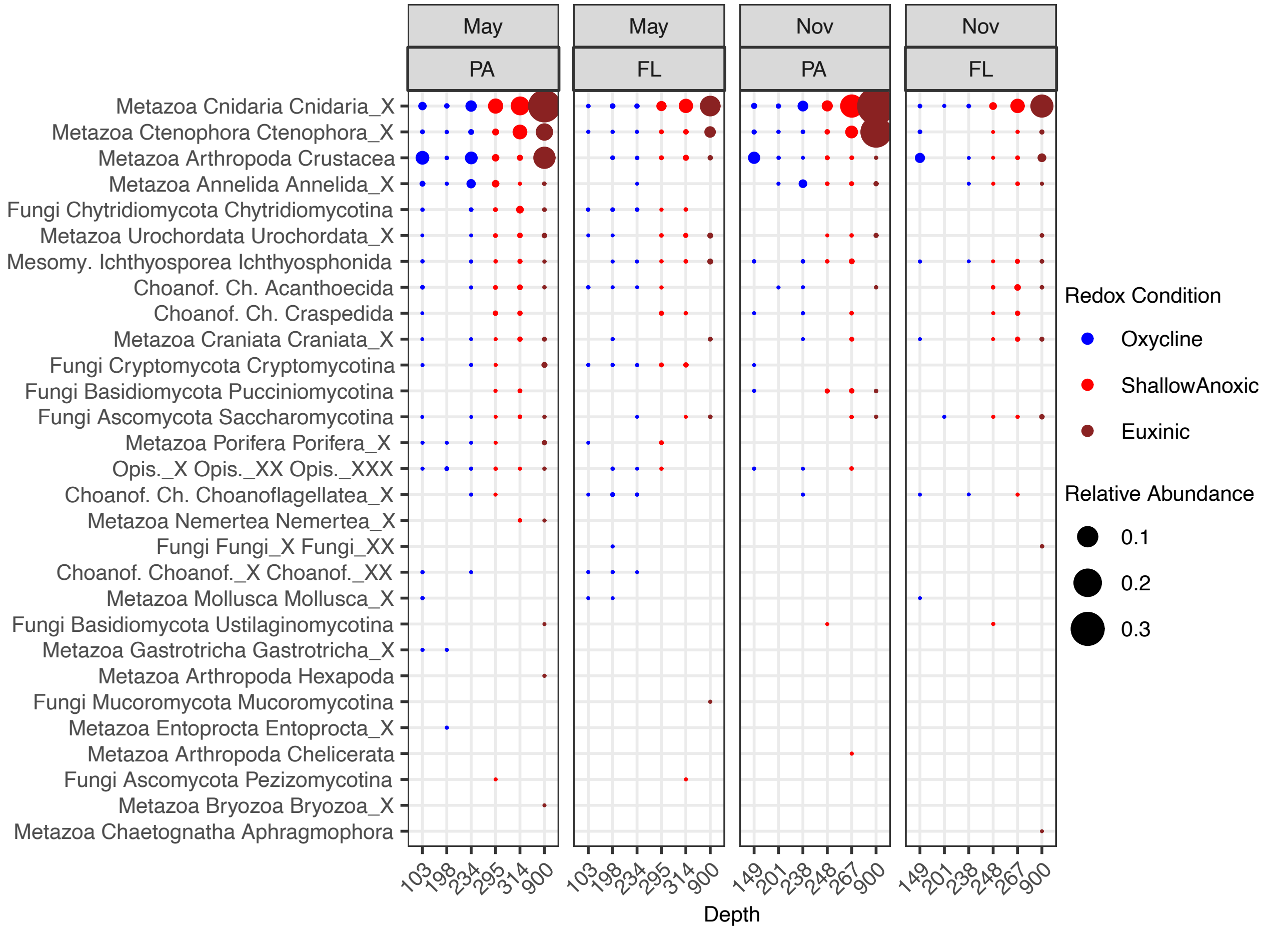

Figure S3: Relative abundance of 18S rRNA ASVs within the Opisthokonta supergroup from May 7-9, 2014 and November 5-7, 2014 cruises and both size fractions (PA and FL). Taxa were binned at the level of Order. Some taxa names were shortened to save space: Choanof = Choanoflagellida; Ch = Choanoflagellatea; Mesomy = Mesomycetozoa; Opis = Opisthokonta.

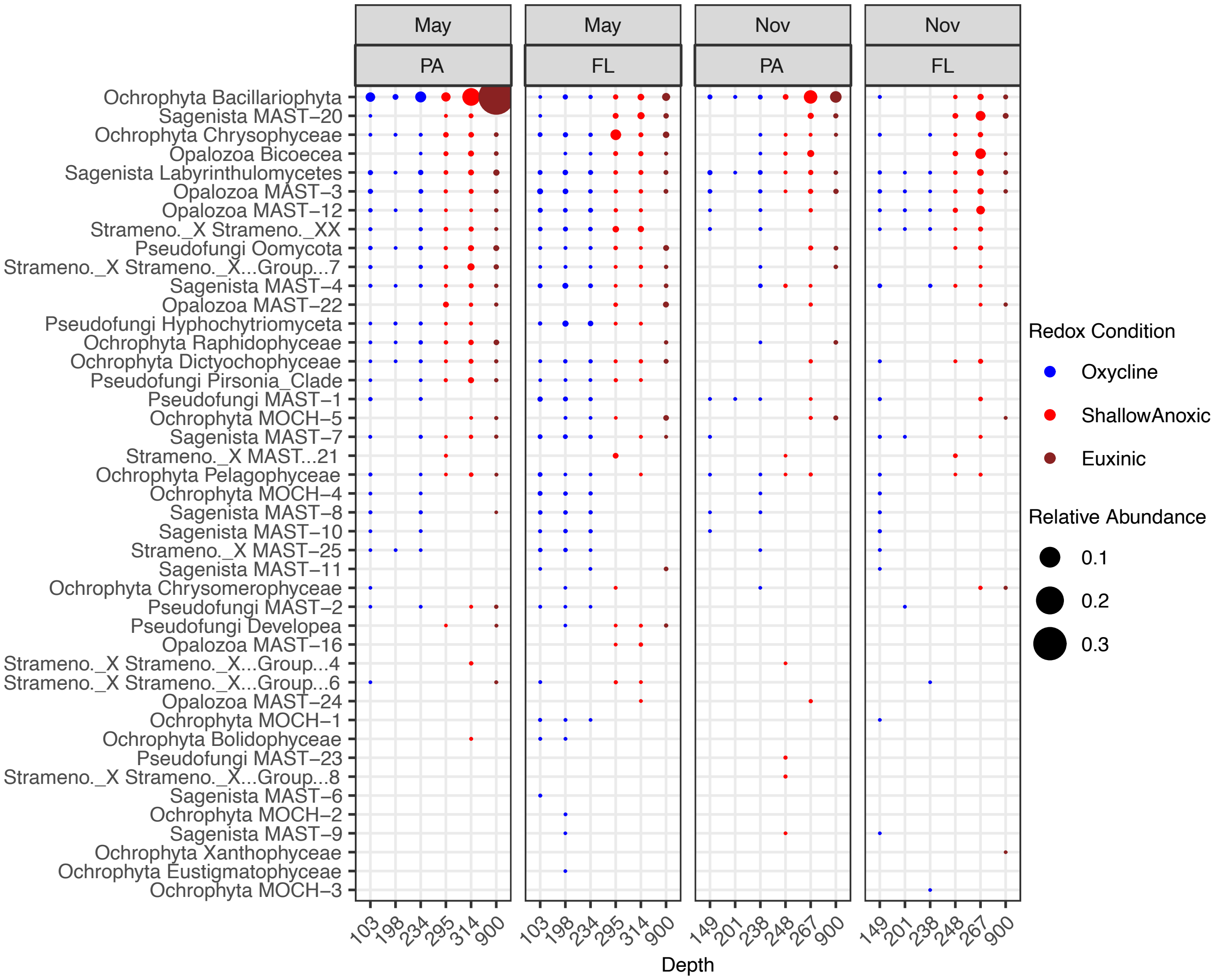

Figure S4: Relative abundance of 18S rRNA ASVs within the Stramenopile superphylum from May 7-9, 2014 and November 5-7, 2014 cruises and both size fractions (PA and FL). Taxa were binned at the level of Class. Some taxa names were shortened to save space: Strameno = Stramenopiles;

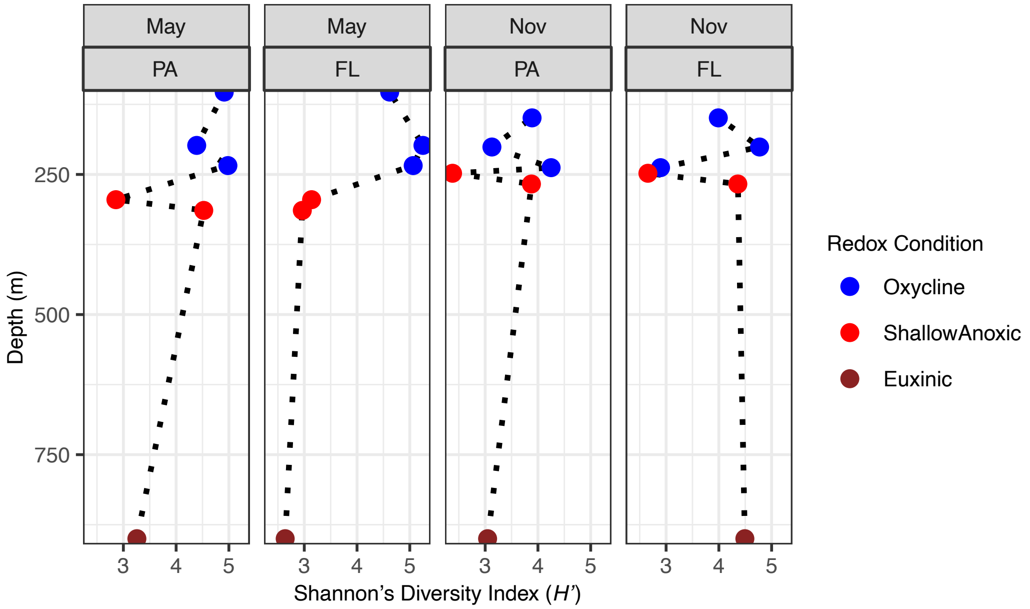

Figure S5: Shannon's diversity index (*H'*) based on eukaryotic ASVs. Points represent means of duplicate libraries replicates.

Table S1: A list of the significantly correlated ASVs from the ordination analysis and their PC1, PC2, r^2, and p-values from Fig. 5D.

*(S*upplemental file: "*Table 1.xlsx")*

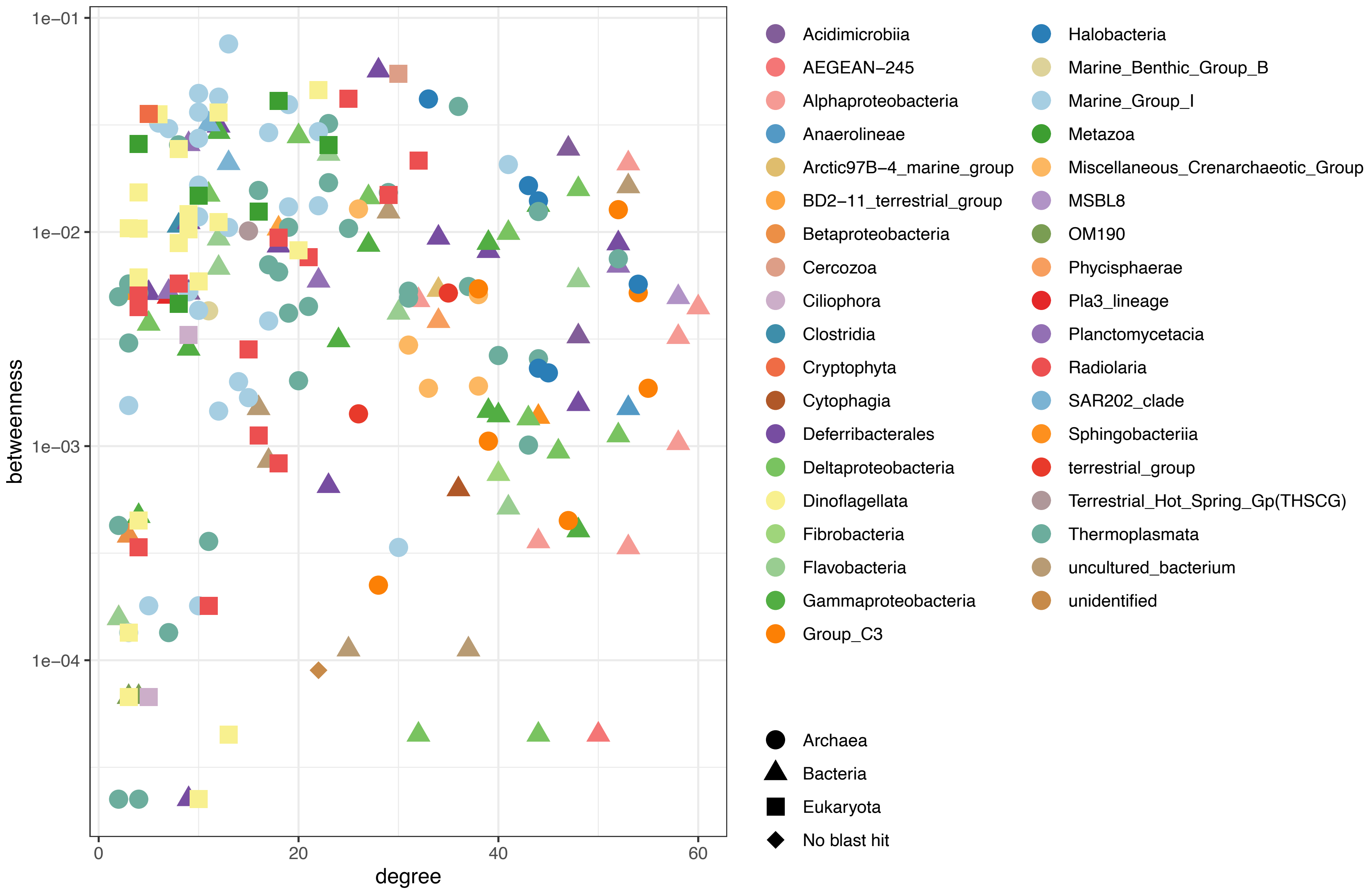

Figure S6: Betweenness centrality and node degree of each node from the SpiecEasi network calculated from the full dataset. Nodes are color-coded by taxonomy and shape indicates domain.
